# 

**Published:** 1823-03-01

**Authors:** 


### BIBLIOGRAPHICAL RECORD;

OR,

*Works received for Review since last Quarter.*

1. A Treatise on the Disease termed PUERPERAL FEVER; illustrated by numerous Cases and Dissections. By JOHN MACKINTOSH, M. D. One vol. 8vo, pp. 323. Edinburgh. Nov. 1822.

---

2. A Historical and Topographical Essay upon the Islands of Corfu, Leucadia, Cephalonia, Ithaca, and Zanté; with Remarks upon the Character, Manners, and Customs of the Ionian Greeks, &c. By WILLIAM GOODISON, A. B. Assistant Surgeon in His Majesty's 75th Regiment. Octavo, pp. 267, with numerous maps and sketches, price 12s. 6d. boards. October, 1822.

☞ *We recommend this interesting little volume to all officers, civil and military, going to the Mediterranean, as containing a great mass of entertaining information. The medical matter will be found in the next number of our Journal.*

---

3. A Treatise on the Epidemic Puerperal Fever, as it prevailed in Edinburgh in 1821-22. To which is added an Appendix, containing the Essay of the late Dr. Gordon on the Puerperal Fever of Aberdeen in 1789-90-91-92. By WILLIAM CAMPBELL, M. D. Fellow of the Royal College of Surgeons; one of the Medical Officers of the Royal Public Dispensary, Lecturer on Midwifery. Octavo, pp. 400. Edinburgh and London, 1822.

---

4. Observations on the Acute and Chronic Dysentery of Ireland; containing a Historical View of the Progress of the Disease in Ireland, with an Enquiry into its Causes; and an Account of its Symptoms and Mode of Treatment; with a Report of selected Cases. By JOHN O'BRIEN, M. D. Fellow and Censor of the King and Queen's College of Physicians in Ireland; and Physician to the Fever Hospital and House of Recovery, Cork Street, Dublin. Octavo, pp. 100. Dublin, 1822.

☞ *To be reviewed in our next along with some other works on the same subject.*

---

5. Illustrations of the Enquiry respecting Tuberculous Diseases. By JOHN BARON, M. D. Physician to the General Infirmary at Gloucester. Octavo, pp. 234, and five coloured plates. London, 1822.

---

6. A Description of the Human Muscles, with their several Uses, and the Synonyma of the best Authors. By JOHN INNES. A new Edition, with Notes, practical and explanatory, by JOHN HUNTER, Surgeon and Lecturer on Anatomy in Glasgow. Illustrated with 18 new engravings of the muscles, by W. H. Lizars; 12mo, pp. 180. London & Glasgow, 1822.

☞ *Innes's Description of the Human Muscles has long held a distinguished place among our English medical classics. Mr. Hunter has improved the original Work by the insertion of practical and explanatory notes, which seem to us to be judiciously selected and perspicuously expressed. On this and other grounds, therefore, we can recommend his edition of INNES, as a cheap and faithful guide to students in the course*

*of their dissections, and even to the operative surgeon, when preparing himself to relieve or eradicate diseases with the knife. Did our space admit quotations, we should transfer to our own columns the notes standing at pages* 30, 42, 45, 53, 75, 95, *and* 115, *with others of equal importance. The plates are equal to any of the kind we have seen : the volume, indeed, is well worthy the attention and patronage of those for whose use it has been prepared.*

---

7. Practical Observations on the Treatment and Cure of several Varieties of Pulmonary Consumption ; and on the Effects of the Vapour of Boiling Tar in that Disease. By Sir ALEXANDER CRICHTON, M.D. F.R.S. Physician in Ordinary to their Imperial Majesties the Emperor and Dowager Empress of Russia, to His Royal Highness the Duke of Cambridge, &c. &c. One volume, octavo, pp. 261. London, December 1822.

---

8 Sketches of Field Sports, as followed by the Natives of India, with Observations on the Animals. Also, an Account of some of the Customs of the Inhabitants, a Description of the Art of catching Serpents, as practised by the *Cunjoors*, and their Method of curing themselves when bitten ; with Remarks on Hydrophobia and Rabid Animals. By DANIEL JOHNSON, formerly Surgeon in the Honourable East India Company's Service, and many years resident at Chittrah in Ramghur. Octavo, pp. 261, and one plate. London, December 1822.

☞ *This little volume contains a great mass of local information, which will be found most useful as well as amusing to the young civil, military, and medical officer in the East Indies. To them we recommend it conscientiously. It will also be found to contain much entertaining matter for the general reader in this country. We shall give some extracts from the medical portion of it in an early Periscope.*

---

9. Pharmacopœia Imperialis, sive Pharmacopœia Londinensis, Edinburgensis, et Dublinensis collatæ ; cum notis Anglicis Decompositiones Chemicas Exponentibus. Small 8vo, pp. 255. London, Dec. 1822.

☞ *The Collator's preface will best explain the nature of this little work.*

*" The design of the Pharmacopœia Imperialis is to give a comparative view of all the formulæ in the last editions of the three Pharmacopœias, with a brief explanation of those processes in which the chemical changes produced are most worthy of remark. The Latin text has accordingly been preferred, and the corresponding formulæ have been successively arranged, so as to afford the best means of comparing them. In selecting, among several methods of accomplishing this design which were suggested, it was finally resolved to follow the plan of the London College, and to insert the formulæ of the Edinburgh and Dublin Colleges in their proper places conformable to this method. The Chemical Remarks have been made as short as was judged to be consistent with perspicuity : had these been more ample and copious, the book would have been rendered more expensive, without, perhaps, adding very materially to its value."—Preface.*

*The work is materially different from the Conspectus of Mr. Thompson, inasmuch as it gives the whole of the formulæ and directions of the three Pharmacopœiæ at full length, in Latin. It does not, however, give the properties, doses, uses, &c. of the medicines, as in Mr. T's Conspectus.*

---

10. An Essay on the Medicinal Efficacy and Employment of the Bath Waters, illustrated by Remarks on the Physiology and Pathology of the Animal Frame, with reference to the Treatment of Gout, Rheumatism,

Palsy, and Eruptive Diseases. By EDWARD BARLOW, M.D. Graduate of the University of Edinburgh, Member of the Royal College of Surgeons of Ireland, one of the Physicians of the Bath Hospital, &c. Octavo, pp. 200. December 1822.

---

11. Memoria su di un'Operazione di Litotomia Digna di Particolare Considerazione, &c. Da ANTONIO TRASMONDI ROMANO, &c. Quarto, pp. 27. Roma, 1822.

☞ *We return thanks to Mr. Babington for the above.*

---

12. History and Method of Cure of the various Species of Epilepsy: being the Second Part of the Second Volume of a Treatise on Nervous Diseases. By JOHN COOKE, M.D. F.R.S. F.A.S. &c. Cctavo, pp. 235. Longman and Co. February 1823.

---

### XV.

### INTELLIGENCE, CORRESPONDENCE, &c.

### *List of Subscribers since last Quarter.*

Archibald, Mr. Charles—to South America
Armstrong, Dr. Dublin
Allardice, Mr. Jas. Surg. Stokesby
Anderson, Alexander Dunlop, Esq. C. M. Glasgow
Benjamin, W. Esq. Surgeon
Black, Mr. Surgeon, Exmouth
Burns, John, Esq. C.M. Profes. of Surgery, University of Glasgow
Barker, James, Esq. Colchester
Chaldecott, Mr. Surg. Dorking, Surrey
Cochrane, Harman, M. D.
Canham, Dr. (misstated Mr.) Stevenage
Calonne, Dr. Dublin
Crawford, William, Esq. Surgeon, Glasgow
Catlaw, Mr. Thornton, Yorkshire
Doughty, Mr. Surgeon, Goodge Street, Tottenham Court Road
Dilworth, Mr. Edward, (Student)
Durie, Dr. Dublin
Dennis, P. Esq. Surgeon, Northumberland Militia
Davis, Mr. J. B.
Dodshaw, R. Esq. M. R. C. S. Norfolk
Edmunds, Mr. John, Surgeon, Wolston, Coventry
Edwards, Henry, Esq. Newgate Street
Farmer, Mr. Surgeon, Dorset Street, Portman Square
Francis, Mr. J. O. Surgeon, Ipswich
Fielding, Mr. George, Surgeon, Hull
Fleminge, Mr. Surgeon, Kentish Town
Fulton, Dr. Dublin
Greenfield, Mr. Frederick, Surg. Poland Street
Harrison, Dr. Holles Street, Cavendish Square
Harty, Dr. Dublin
Hunter, Dr. Dublin Castle
Hornby, Mr. Thomas, Taxford
James, ——, Esq. Surg. Croydon
Kinchela, Dr. Dublin
Kennedy, Dr. Dublin
Lickorish, Dr. Rich. Wolston, Coventry
Moran, P. Esq. Sheerness
Morton, Edward, M. B. Member of Trinity College, Cambridge, Lower Eaton Street, Pimlico
Morgan, Mr. William Francis, Surgeon, Bristol
Maysmor, Mr. H. Surgeon, Bengal Establishment
Mossman, Dr. Bradford, Yorkshire
Norfolk Medical Book-Society, 2 copies
Nicholson, Dr. Dublin
Oxley, Mr. Chas.—to West Indies
Propert, Mr. Surg. Duke Street, Portland Place
Peck, Mr. Surgeon, Newmarket
Quinn, Dr. Dublin
Robertson, Robert, Esq. Glenarback
Seeds, Mr. George, Surg, Portsea
Scarman, Mr. Madras Establishment
Smart, Mr. G. Hutton Bushell, York
Vineash, Mr. Surg. Poland Street
Vaux, George, Esq.
Ward, Thomas, M.D. Assist. Surg. East India Company's Service
Wilson, James, Esq. Surgeon, H. M. S. Gloucester—to West Indies
Willsher, Mr. Thos. Steele, Fellow of the Royal College of Surgeons of London, Weathersfield, near Braintree, Essex
Wylde, Mr. Surgeon, Middenham
White, Surgeon, Dublin
Worts, William, Esq. M.R.C.S. &c. Colchester
Woolley, Mr. J. Brompton
Waller, Mr. Luton

☞ We regret that we have been obliged to postpone the *general* list of subscribers till the close of next volume.

REMOVAL.

M. Richard Maysmor, from Ashburn to Teddington, Middlesex.

### INDEX.

| | Page |
|---|---|
| Aneurism of the heart, curious case of | 20 |
| Asthma, Dr. Parry's pathology of | 20 |
| Abstinence, remarkable case of | 49 |
| Antimoniated ointment, Dr. Jenner on | 89 |
| Anasarcous limbs, puncture of, | 182 |
| Ascites, cured by pressure | 189 |
| Arteritis | 194 |
| Arsenic, on the poison of | 203 |
| Aristotle on life | 250 |
| Abernethy on life | 253 |
| Axillary aneurism, operated on | 402 |
| Aneurism, axillary, cured | 402 |
| Anatomy, surgical, Mr. Anderson's | 357 |
| Anderson, Mr. on surgical anatomy | 357 |
| Axillary aneurism, Mr. Mayo's case of | 390 |
| Ascites, remarkable case of | 425 |
| Acid bath, Mr. Coyne on | 510 |
| Apoplexy, Morgagni's remarks on | 548 |
| Arteries, non-pulsation of | 566 |
| Alibert, Dr. on prurigo formicans | 779 |
| Angina pectoris, various cases of | 809 |
| Amesbury, Mr. on fractures of the thigh, &c. | 916 |

B.

| | Page |
|---|---|
| Barlow, Mr. on surgery and midwifery | 64 |
| Bistouri, Cachée, proposed by Mr. Barlow | 70 |
| Blicke, Dr. on cephalitis | 178 |
| Bath-waters, on | 188 |
| Bronchocele, case of, operated on | 194 |
| Breschet, M. on melanose | 208 |
| Barclay, Dr. on life, &c. | 225 |
| Blumenbach, remarks on | 236 |
| Barlow, Mr. on the placenta | 306 |
| Boyer, M. on the sphincter ani | 328 |
| Belladonna, observations on | 412 |
| Blood, foreign matters in | 416 |
| Brain, remarkable wound of | 428 |
| ——— Pathology of, in mania | 434 |
| ——— M. Lallemand on diseases of | 465 |
| Bourne, Dr. case of cardiac disease | 571 |
| Blane, Sir Gilbert, dissertations | 783 |
| Brera, Professor, on iodine | 757 |
| Bladder, Mr. Bingham on | 767 |
| Baron, Dr. on tuberculous diseases | 785 |

C.

| | Page |
|---|---|
| Carotids, effects of pressure on | 26 |
| Conversion of diseases | 32 |
| Ceylon, Mr. Marshall on | 53 |
| Clarke, Mr. on discharges of females | 77 |
| Carcinoma uteri, Mr. Clarke on | 85 |
| Corroding ulcer of the uterus | 81 |
| Chisholm, Dr. on tropical climates | 100 |
| Climate of West Indies, Dr. Chisholm on | 100 |
| Chronic hepatitis, Dr. Chisholm | 119 |
| Clark, Dr. Letter to Tommasini | 151 |
| Clinical school of Edinburgh | 153 |
| Cephalitis, Dr. Blicke on | 178 |
| Cornish, Mr. on dislocated femur | 180 |
| Carson, Dr. on phthisis | 183 |
| Chagrin, influence of on the liver | 202 |
| Cuvier, remarks on | 238 |
| Cabanis, remarks | 242 |
| Craniology, remarks on | 245 |
| Cæsarian operation, successful | 324 |
| Constriction, of the sphincter ani | 328 |
| Carcinoma, mistaken | 420 |
| Cancer of the lip, M. Richerand on | 427 |
| Counter irritation | 429 |
| Chlorine, Mr. Wallace on | 510 |
| Coyne, Mr. on the nit. mur. acid bath | 510 |
| Cooke, Mr. his abridgment of Morgagni | 546 |
| Clutterbuck, Dr. case of cardiac disease | 570 |
| Cynanche cellularis | 644 |
| Chorea, remarkable case of | 659 |
| Cooper, Sir Astley, on dislocations, &c. | 612, 832 |
| Cystitis, Mr. Liston's case of | 681 |
| Cancer of the lip, operation for | 696 |
| Catarrhus vesicæ, observations on | 769 |
| Clavicle, dislocations of | 838 |

Page

D.

Dropsy, final causes of........ 16
Determination of blood, what? 18
———— defective, what? 36
Dyspepsia, nature of.......... 21
Dropsy, curious termination of 53
Dysentery, observations on.... 63
———— Dr. Chisholm on .. 112
Dropsy, inflammatory case of .. 200
Diabetes, remarks on ........ 265
Dubois, Mr. on malignant ulcer of penis ................ 405
———— on inguinal hernia 410
Dyspepsia, congenital ........ 407
Digitalis, over-dose of ........ 425
Danglison's translation of Mosca 534
Dropsy, renal, curious cases of 653
Dislocations, Sir Astley Cooper on ...................... 612
Dunn, Mr. on compound fractures ...................... 669
Distortions, spinal ........... 746

E.

Epilepsy, remarks on .......... 29
Eruptions, artificial, Dr. Jenner on ...................... 89
Epilepsy, Dr. Prichard on ..... 127
Edinburgh medical school defended.................... 151
———— surgical school, remarks on ................ 207
Esquirol on puerperal mania .. 269
Enteric mania, description of.. 291
Emetics in constipation ...... 410
Emetic tartar, Mr. Jeffreys on 539
Elm bark, Mr. Jeffreys on ..... 544
Epliepsy, Morgagni on......... 554
Earle, Mr. Henry, galvanic experience................. 608

F.

Fever, yellow, Dr. Chisholm on 109
Fielding, Mr. on fractured patella...................... 179
Femur, on dislocation of ...... 180
Femoro-coxalgia, moxa in.... 530
Forbes, Dr. on variolous epidemic ............ 646
———— on tar vapour ..... 651
Fractures of the thigh bone.... 628
Fosbroke, Mr. case of scrofula 671
Fractures, compound, Mr. Dunn on ...................... 669
Fœtus extracted post mortem.. 677
Fever, the various theories of .. 818

G.

Goitre, connected with cardiac disease.................... 18

Page

Gout, a salutary process ...... 38
Gibney, Dr. on oil of turpentine 406
Geneva, salubrity of.......... 383
Good, Dr. J. M. study of medicine................. 574, 806
Gregory, Dr. on cynanche cellularis...................... 644
Gooch, Dr. on uterine hæmorrhage ..................... 662
Georget, Dr. on mania......... 701

H.

Hæmorrhage, final causes of .. 16
Hygiene, tropical ............ 105
Hepatic dysentery............. 113
Hepatitis, Dr. Chisholm on.... 115
Hepatic epilepsy ............. 146
Hill. Mr. on tic douloureux.... 176
Harvey on life .............. 251
Hunter on life .............. 252
Hippocrates, critique on ...... 270
Hip-joint, scrofula of ........ 340
Hernia, Mr. Lizars on........ 408
Heart, venereal affection of.... 413
Hip-joint, luxation of ........ 415
Hall, Dr. Marshall, cases by.... 387
Hydrophobia ................ 429
Hare-lip, double, curious case of 433
Hutchinson, Mr. on neuralgia.. 495
Hydrophobia, observations on.. 559
Heart, remarkable disease of .. 563
Hunter, John, remarks on his death ..................... 568
Helminthia, Dr. Good on...... 585
Hæmorrhoids, Dr. Good on.... 588
Hip-joint dislocation, Sir Astley Cooper .................. 620
Hypochondriasis, curious case of 667
Hepato-pulmonic disease...... 675
Hurricane, causes of.......... 743

I.

Inflammation, final causes of.. 16
Inflammation, remarkable case of ...................... 197
Iliac artery, obliteration of.... 431
Iris, on the powers of the...... 533
Icterus, pathology of.......... 589
Iritis, Dr. Smith on.......... 666
Iodine, Dr. Brera, &c. on...... 757

J.

Jenner, Dr. on artificial eruptions ..................... 89
Jaundice, remarkable case of .. 96
Jeffries, Mr. cases in surgery.. 535
Johnson, Dr. James, on renal dropsy.................... 655

Page

K.

Kandyan country, topography of ........ 55
Kennedy, Dr. on tetanus...... 423
Kidney, dropsy of............ 653
Knee, dislocations of ......... 832

L.

Lithotomy, Mr. Barlow on .... 65
Ligature of arteries, described by Celsus ................ 68
Lithotomy, remarkable case of 73
Laird's, Dr. case of hooping-cough .................. 131
Lloyd, Mr. on scrophula .. 156, 333
Liston, Mr. his letters respecting the Infirmary of Edinburgh .................. 207
Life, Dr. Barclay on .......... 225
Lucretius, remarks on........ 232
Lawrence, Mr. remarks on .... 239
Labour, premature, on.. .... 319
Lizars, Mr. on hernia ........ 408
Lallemand, M. on softening of the brain.................. 465
Larrey, Baron, surgical memoirs 523
Liver, remarkable disease of .. 694

M.

Marshall, Mr. on Ceylon ...... 53
Mammary scrophula.......... 169
Melanose, M. Breschet on .... 208
Materialism.................. 226
Magnetism, animal .......... 255
Mania, puerperal, M. Esquirol on .................. 269
——— Dr. Prichard on ...... 277
Metastasis, curious cases of.... 401
Malaria in St. James's Park.... 422
Medico-chirurgical transactions, vol. xii.................. 386
Mayo, Mr. case of axillary aneurism.................. 390
Moxa, Baron Larrey on ...... 523
——— Mr. Dunglison on ...... 534
Mammary abscess, treatment of 541
Morgagni, abridged by Cooke.. 546
Medicine, the study of, Dr. Good's.................. 574
Melena, Dr. Good on ........ 592
Metastatic inflammations ..... 641
Magendie, Dr. on Chorea ..... 659
Mania, M. Georget on........ 701
Macmichael, Dr. on scarlet fever 724
Mortality, comparative, in London .................. 737

N.

Nervous constitution, Dr. Parry on .................. 25
Nose, remarkable tumour on.. 76
Nervous system, Dr. Prichard on .................. 122
Nimmo, Dr. on oil of Croton .. 186
Neuralgia, various authors on.. 495
Nostalgia, Baron Larrey on.... 532
Non-pulsation of arteries...... 566
Nerves spinal, experiments on . 682

O.

Obstruction of the bowels, cases of.................. 51
Oil of croton-tiglium ........ 186
Ovarian dropsy cured by operation .................. 190
Organization and life......... 225
Oil of turpentine, Dr. Gibney on 406
Opium, on poisoning by ...... 433
Ophthalmia, scrophulous, Mr. Jeffreys .................. 535
Œsophagus, stricture of ...... 540
Dito ditto ......... 677

P.

Parry, Dr. Elements of pathology .................. 1
Pathology, Dr. Parry's, Elements of.................. 1
Pulsations in epigastrio, what.. 22
Prostate gland, remarkable enlargement of .............. 74
Purulent discharges, Mr. Clark on .................. 77
Prichard, Dr. on the nervous system.................. 122
Pathology of epilepsy ......... 129
Prostatic scrophula........... 173
Patella, on fracture of ........ 179
Paralysis (nervous) case of .... 181
Phthisis, proposal in.......... 183
Pulmonary disease, obscure case of.................. 185
Poisoning by arsenic.......... 192
Phrenology, remarks on ...... 245
Puerperal mania, Esquirol on.. 269
Prichard, Dr. on mania ...... 277
Pathology of the brain in mania 282
Placenta, Mr. Barlow on...... 306
Premature births ............ 320
Pelvis, distortions of......... 318
Phlegmasia dolens, Dr. Hosack on .................. 432
Pathology of the brain, M. Lallemand .................. 465
Palmer, Dr. S. on neuralgia ... 495
Philip, Dr. Wilson, his physiology .................. 596
Physiology, general principles of 596
Puerperal fever, Mr. Moir on .. 648
Pregnancy, simulated ........ 652

| | Page |
|---|---|
| Puerperal fever, Dr. Payne.... | 678 |
| Purpura hæmorrhagica ........ | 679 |
| Poison, apparatus for removing | 683 |
| Prurigo formicans, M. Alibert on .................... | 779 |
| Patella, fractures of .......... | 834 |
| Phrenology, observations on ... | 896 |

R.

| | |
|---|---|
| Rectum, carcinoma of ........ | 84 |
| Remittent, or yellow fever .... | 109 |
| Rectum, Mr. White on........ | 326 |
| Rheumatism, chronic .......... | 406 |
| Receuil de memoires de chirurgie .................... | 523 |
| Rickets, Moxa in ............ | 529 |
| Rectum, stricture of.......... | 587 |
| Retention of urine............ | 773 |
| Respiratory function.......... | 806 |

S.

| | |
|---|---|
| Small-pox, on the antiquity of.. | 41 |
| Smith, Dr. Ashby, his edition of Willan.................... | 41 |
| Seneca's description of small-pox .................... | 46 |
| Surgery, Mr. Barlow on...... | 64 |
| Scrophula, Mr. Lloyd on .. 156, | 333 |
| Smerdon, Mr. on arteritis ..... | 196 |
| Spine, scrophula of .......... | 342 |
| Strammonium in rheumatism.. | 406 |
| Spasmodic diseases, Dr. Moulson on ...................... | 555 |
| Study of medicine, Dr. Good's.. | 574 |
| Stricture, spasmodic, of the rectum ...................... | 586 |
| Stethescope in pregnancy ..... | 661 |
| Suicide, tendency to .. 671, 672, | 684 |
| Spinal distortion ............. | 686 |
| Scarlet fever, Dr. Macmichael on ...................... | 724 |
| Spine, Mr. Ward on .......... | 746 |
| Sternalgia, or angina pectoris.. | 809 |
| Shoulder, dislocations of ...... | 840 |

T.

| | |
|---|---|
| Thyroid gland—a diverticulum? | 20 |
| Tic douloureux, remarks on ... | 30 |
| Topography, medical, of Ceylon | 53 |
| Tropical climates, Dr. Chisholm on ...................... | 100 |
| Tommasini on English medicine | 151 |
| Testicular scrophula.......... | 170 |
| Tic douloureux .............. | 176 |
| Tetanus, case cured .......... | 181 |
| Tympanites, intestinalis ...... | 193 |
| Todd, Mr. on axillary aneurism | 402 |
| Tobacco, in the phlegmasiæ.. | 414 |
| Tetanus, traumatic .......... | 423 |
| Tongue, ulcerations of ........ | 543 |
| Tar vapour, Dr. Forbes on .... | 651 |
| Tartrite of antimony, large doses | 680 |
| Tic douloureux, Dr. Wilson on . | 691 |
| Tuberculous diseases, Dr. Baron on ...................... | 785 |
| Tibia, dislocation of.......... | 833 |
| Thigh, Mr. Amesbury on fractures of.................... | 915 |

U.

| | |
|---|---|
| Uterine inflammation ........ | 78 |
| Uterine epilepsy ............. | 132 |
| Urinary organs, Mr. Wilson on | 260 |
| Urine, suppression and retention of ...................... | 265 |
| Uterine mania .............. | 285 |
| Ulcer, malignant, of the prepuce | 405 |
| Uterus, remarkable contraction of...................... | 425 |
| Uterine hæmorrhage, Dr. Gooch on ...................... | 662 |
| Uterus, retroversion of........ | 673 |
| Uvula, elongated ............ | 681 |

V.

| | |
|---|---|
| Vessels, erectile powers of.... | 24 |
| Vaginal inflammation ........ | 79 |
| Variola and varicella, identity of | 198 |
| Voltaic battery .............. | 206 |
| Vomiting, effects of in apoplexy | 550 |
| Variolous epidemic, Dr. Forbes on ...................... | 646 |

W.

| | |
|---|---|
| Willan, Dr. his miscellaneous works .................... | 41 |
| Wilson, Mr. on the urinary organs.......... | 260 |
| ————account of his death | 267 |
| White, Mr. on the rectum .... | 326 |
| Wallace, Mr. on chlorine...... | 510 |
| Ward, Mr. on spinal distortions | 746 |

Y.

| | |
|---|---|
| Yellow fever, Dr. Chisholm on | 109 |
| ———— Mr. Coventry on | 184 |
| Yeats, Dr. G. D. on neuralgia.. | 495 |
| Yellow fever, Sir Gilbert Blane on ...................... | 739 |

### CLASSIFIED INDEX.

Page.

### I.

### *HEAD.*

Cancer of the lip .......... {427, 696}

#### 1.—*Brain.*

Epilepsy, Dr. Prichard on...... 127
Cephalitis .................. 178
Physiology of Life........... 225
Mania, Dr. Prichard on ...... 277
Remarkable wound of the brain 428
Pathology of the brain in mania 434
Lallemand, on the brain and appendages.................. 465
Morgagni on Apoplexy, &c..... 548
Hypochondrias and suicide .. {667, 672, 684}
Mania, Dr. Georget on........ 701
Phrenology, observations on.... 896

#### 2.—*Coverings of the Brain.*

Lallemand on the coverings of the brain.................. 465

#### 3.—*Spinal Marrow and Envelopes.*

Experiments on the spinal nerves 682

#### 4.—*Organs of Sense.*

Scrophulous Ophthalmia...... 536
Iritis, Dr. Smith on .......... 666

### II.

### *NECK.*

Bronchocele ................ 194
Inflammation of the glottis.... 387
Carcinoma in the neck........ 420
Stricture of the œsophagus .... 540
Cynanche cellularis .......... 644
Strictured œsophagus ........ 677
Uvula, elongated ............ 681

Page.

### III.

### *CHEST.*

#### 1.—*Lungs and Coverings.*

Phthisis .................. 183
Pulmonary disease............ 185
Phthisis, Dr. Chisholm on .... 379
——— tar vapour in........ 651

#### 2.—*Heart and Coverings.*

Venereal affection of the heart.. 413
Remarkable disease of the heart, &c. .................... 456
Cardiac inflammation ........ 563
Diseases of the heart ........ 566

### IV.

### *ABDOMEN.*

Tympanites ............... 193
Ascites.................... 425

#### 2.—*Stomach.*

Congenital dyspepsia.......... 407
Digestive functions, Dr. Good on 576

#### 3.—*Intestines.*

Fatal constriction of the colon .. 51
Dysentery, Mr. Marshall on.... 62
——— Dr. Chisholm on... 112
Enteric epilepsy ............ 141
Colica Pictonum ............ 376
Worms, Dr. Chisholm on...... 377
Constipation, emetics in...... 410

#### 4.—*Liver.*

Hepatitis, Dr. Chisholm on .... 115
Hepatic epilepsy ............ 146
Hepatic disorder ............ 202
Hepatic affections, chlorine in.. 510
Jaundice, Dr. Good on........ 589
Hepato-pulmonic disease...... 675
Tubercular disease of the liver.. 694

#### 6.—*Kidneys.*

Renal dropsy, cases of ........ 653

### V.

### *PELVIC VISCERA.*

### 1.—*Uterus.*

Page.

Clarke on female discharges .. 77
Uterine epilepsy ............ 132
Ovarian dropsy .............. 190
Utero-gestation mania........ 269
Placenta, Mr. Barlow on ...... 306
Premature delivery .......... 317
Cæsarian operation .......... 324
Contraction of the uterus...... 426
Puerperal fever .............. 648
Apparent pregnancy.......... 652
Auscultation in pregnancy .... 661
Uterine hæmorrhage(Dr.Gooch) 662
Uterus inflamed, retroversion.. 673
Cæsarian operation .......... 678
Puerperal fever .............. 678
Ditto, Drs. Campbell and Mackintosh .................. 858

### 2.—*Bladder and Outlets.*

Urinary organs, Mr. Wilson on 260
Ulcer on the prepuce.......... 405
Cystitis, curious case of ...... 681
Diseases of the bladder, Mr. Bingham on .............. 767

### 3.—*Rectum.*

On strictures of the rectum.... 326

### VI.

### *EXTREMITIES.*

Sir A. Cooper on dislocations { 612, 832

Mr. Dunn on compound fracture 669

### 1.—*Upper Extremities.*

Fractures of the clavicle ...... 211
Dislocations, Sir A. Cooper on.. 840
———— of the shoulder joint...................... 840

### 2.—*Lower Extremities.*

Fracture of the Patella...... { 179, 834
Dislocation of the femur ...... 180
Hernia of the groin ........ { 357, 408
Luxation of the hip joint...... 415
Phlegmasia dolens............ 432
Dislocations of the hip joint (Sir A. Cooper) ............... 620
Fracture of the neck of the femur........ .............. 628

Page.

Dislocation of the knee ...... 832
———— of the tibia ...... 833
Femur, Mr. Amesbury on fractures of .................. 915

### VII.

### *CONSTITUTIONAL DISEASES.*

Elements of pathology, &c. Dr. Parry .................... 1
Death from fanatical abstinence 49
Yellow remittent fever, Dr. Chisholm ............... 109
Tetanus.................. { 181, 209, 423
Fever, Dr. Armstrong on...... 393
Metastases, curious cases of.. 401
Chronic rheumatism.......... 406
Yellow fever, Sir G. Blane on.. 739
Fever, the various theories of.. 818

### 1.—*Nervous System.*

Diseases of the nervous system, Dr. Prichard .............. 122
Tic Douloureux ............ { 176, 691
Nervous Paralysis............ 181
Chorea ...................... 377
Hydrophobia....... ...... { 429, 559
Neuralgia facialis ............ 495
Spinal affections, galvanism in 608
Nervous system, curious disease of........................ 659
Nerves of the spine, experiments on ...................... 682

### 2.—*Vascular System.*

Arteritis ...................... 194
Inflammatory dropsy.......... 200
Axillary aneurism ............ 400
Foreign matters in the blood .. 416
Obliteration of the external iliac 431
Metastatic inflammations...... 641

### 3.—*Absorbent System.*

Lloyd on scrofula ............ 156
Mr. Fosbroke on scrofula...... 671
Iodine as a medicine in scrofula 757

### 4.—*Dermoid System.*

Small pox, measles, and scarlatina ...................... 41
Jenner on artificial eruptions .. 89
Vaccination ................ 213
Variola and vaccina (Dr. Forbes) .................. 646

| | Page. |
|---|---|
| Scarlet fever, Dr. Macmichael on | 724 |
| Prurigo formicans, Alibert on | 779 |

*Toxicology.*

| | |
|---|---|
| Poisoning by opium | 433 |
| Test for arsenic | 674 |

*Osseous System.*

| | |
|---|---|
| Reunion of entirely separated bone | 400 |
| Dislocations and fractures | 612 |
| Non-union of widely separated bones | 633 |
| Spinal distortions, Mr. Bampfield on | 686 |
| Spinal distortions, Mr. Ward on | 746 |
| ——— dislocations | 852 |
| Mr. Amesbury on fractures of the femur | 915 |

*Medical Agents.*

| | Page. |
|---|---|
| Oil of croton tiglium | 186 |
| Bath waters | 188 |
| Arsenic as a poison | 192, 203 |
| Voltaic battery | 206 |
| Nux vomica | 220 |
| Cubebs and capivi | 221 |
| Oil of turpentine | 406, 446 |
| Emetics in constipation | 410 |
| Belladonna in neuralgia | 412 |
| Opium, over-dose of | 413 |
| Tobacco in the phlegmasiæ | 414 |
| Digitalis, over-dose of | 425 |
| Counter-irritation | 429 |
| Chlorine, and nitro-muriatic acid | 510 |
| Moxa, Baron Larry on | 523 |
| Tartar emetic, Mr. Jeffreys on | 539 |
| Elm-bark as substitute for sarsa | 544 |
| Galvanism in spinal affections | 608 |
| Tar vapour in phthisis | 651 |
| Oil of turpentine in purpura | 679 |
| Tartrite of antimony in Pneumonia | 680 |
| Iodine, various authors on | 757 |

*Surgical Operations.*

| | |
|---|---|
| Lithotomy, Mr. Barlow on | 65 |
| Puncturing anasarcous limbs | 182 |
| Pressure in ascites | 189 |
| Operations for hernia and stone | 357 |
| Ligature of subclavian artery | 390, 400 |
| Operation for lip cancer | 427 |
| ——— extracting poisons swallowed | 683 |

*Medical Topography.*

| | |
|---|---|
| Marshall on the topography of Ceylon | 53 |
| Chisholm on tropical climates | 100 |
| Climate of Geneva | 383 |
| Malaria of London | 422 |

### Notice to Correspondents.

*The Editor begs to say that, when no answer is returned to a correspondent, it may be taken for granted that acquiescence is meant; or that it is not deemed necessary to reply by post. This will save much time to the Editor, and some expence to correspondents.*

*As, in future, no original papers will be published in the extra-limites department, except at the expence of the authors, all information conveyed to the Editor must be considered as at his disposal for incorporation with the analytical reviews. He will, however, whenever a fair opportunity occurs, give full credit, and by name, to the communicator, provided there be no injunction to the contrary.*

*The expence of publishing replies or defences in the extra-limites department is* 9*s.* 6*d. per page; and any number of extra. copies, for the author, may be struck off, for merely the expence of the paper.*

### INSTRUMENT

*For passing a Ligature round a deep-seated Artery,*

INVENTED BY

Mr. WEISS, 62, STRAND, LONDON.

It is well known to the Surgical profession that the most difficult part of an operation for Aneurism is the passing of the ligature round the Artery, if deep-seated, and the extrication of the thread from the eye of the Needle after it is passed round the Artery. The following Instrument, the principle of which was suggested to Mr. Weiss by Mr. Kirby, an eminent Surgeon in Dublin, will be found, it is hoped, to obviate the above-mentioned difficulties. It has already been used with success in Subclavian Aneurism by an eminent Hospital Surgeon of London, and is generally approved of by the Surgical profession.

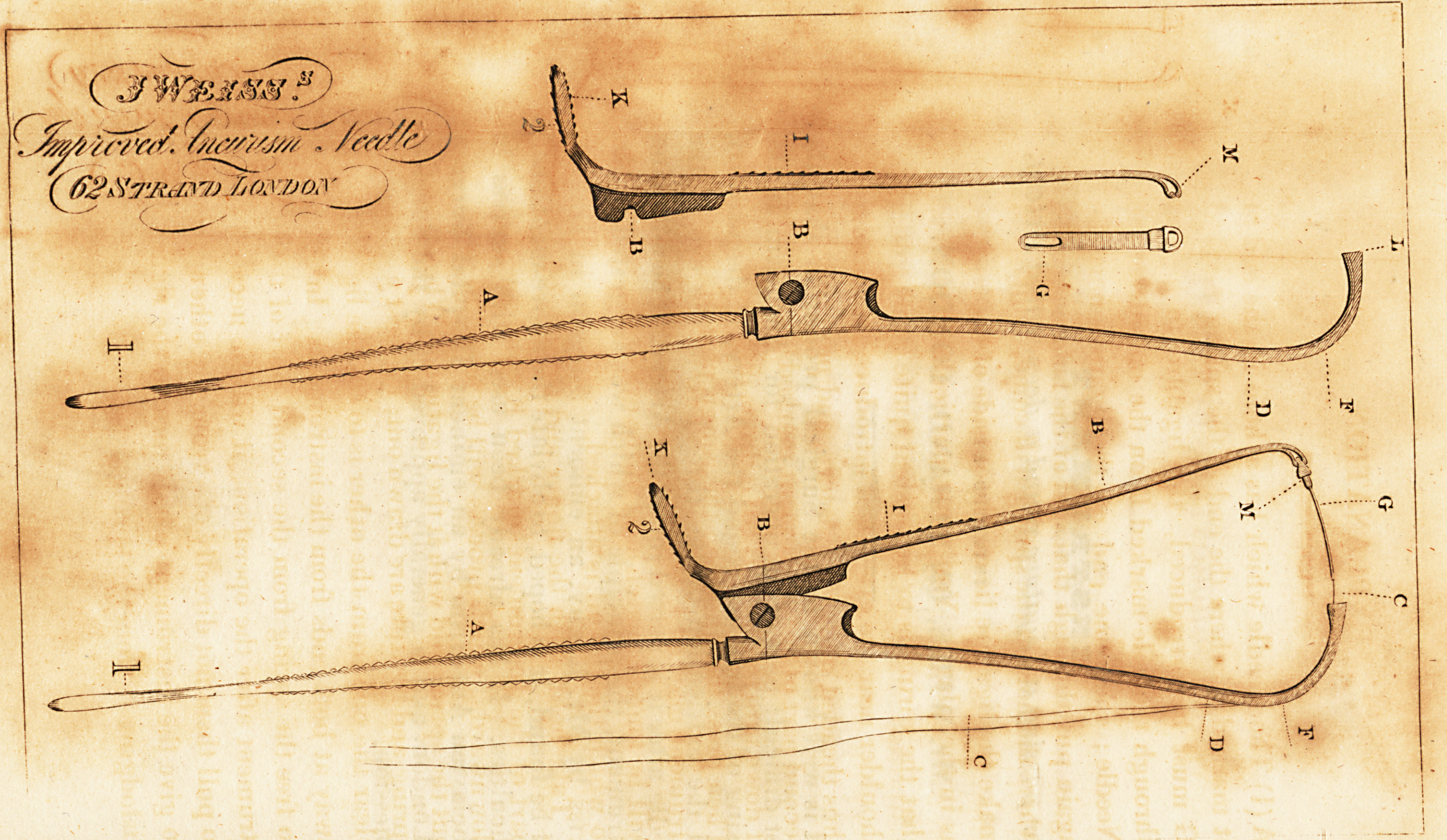

J Weiss's

Improved Aneurism Needle

62 Strand London

### EXPLANATION.

A (1) The Needle which has a hole in the end of it marked L, where the end of the spring marked G must be introduced, the ligature to be put through the hole marked D on the outside of the Needle; the same end of the ligature must be again put through that end of the hole marked F, where the hole in the spring will come, which will make the ligature lie on the outside of the Needle, as in the plate. You must particularly observe that the spring is to be pushed quite home to the shoulder near the eye, then introduce the Needle thus theaded under the Artery, and next take the second part of the instrument marked (2) and lead it down into the joint B till it drops into the screw. Then press the finger on the rough part of the instrument marked I, when the point marked M will be found to catch hold of the eye of the spring to which the thread is attached.

Take hold, with the finger and thumb, of that part of the Instrument marked K, and press it downwards, which process will be found to draw out the spring and with it the ligature. When the spring and ligature are drawn out, the best way to clear the one from the other is to cut the ligature away at both ends from the instrument. In order to free the spring from the second part of the instrument after the operation, it will be necessary to pull them not directly one from the other, but to give the instrument a half turn to the right in dislodging it from the spring.

*New Broad Street, January* 18, 1823.

SIR,

I think it due to your ingenuity to inform you that I yesterday applied a ligature twice to the Subclavian Artery above the Clavicle with your Aneurism Needle, and found its application easy and entirely satisfactory in each instance. Whatever be the result of the Operation, which unforeseen and difficult circumstances render very doubtful, I feel it my duty to state that your Needle removes a difficulty which every operating Surgeon has complained of in his attempts to noose the deep seated Arteries, and that I should be at a loss to name any modern example of the application of a mechanical contrivance to Surgical purposes, so happy in point of simplicity and effect, or so promising in point of usefulness.

I am, Sir,

Yours obediently,

B. TRAVERS.

*Surgeon to St. Thomas's Hospital.*

To Mr. WEISS.

*Observe to turn the Handle by which means you may Dilate to any Extent*

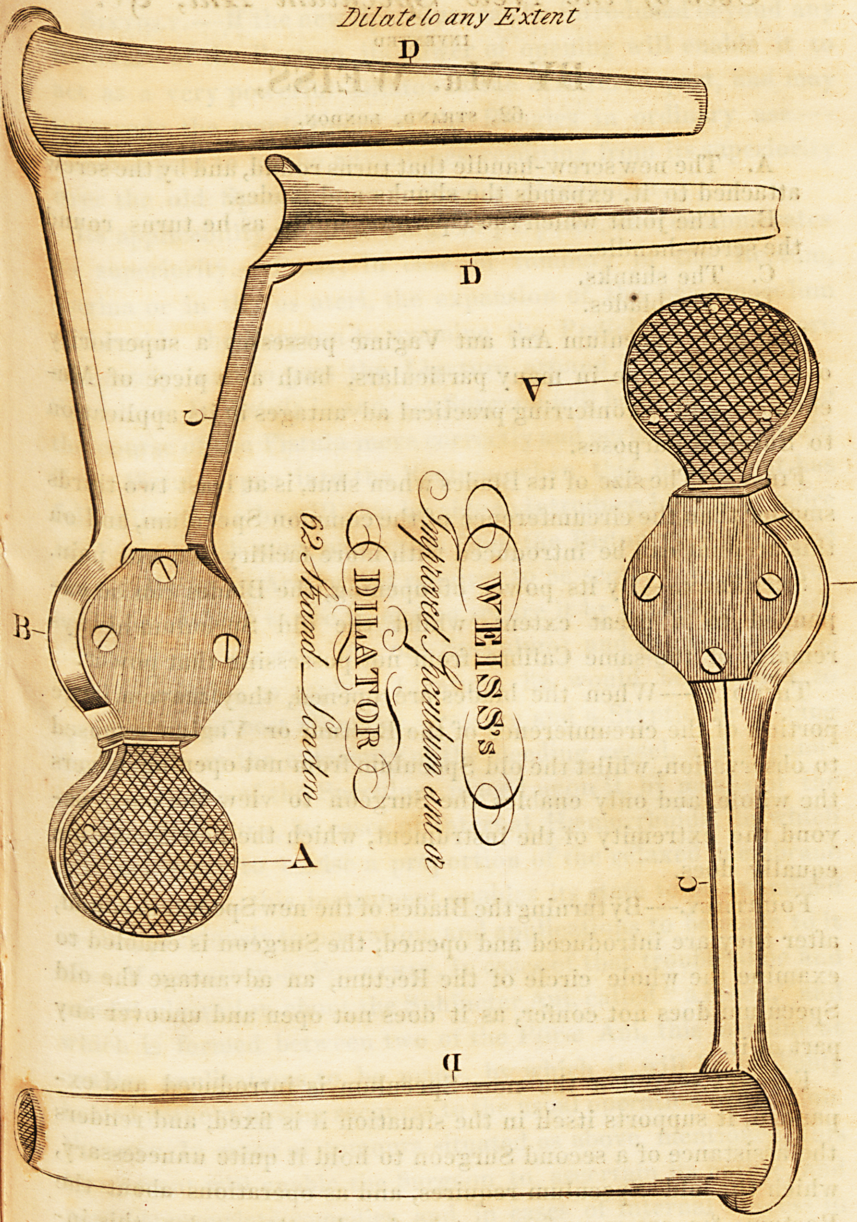

### DESCRIPTION
AND
### *Uses of the New Speculum Ani, &c.*
INVENTED
### BY Mr. WEISS,
62, STRAND, LONDON.

---

A. The new screw-handle that turns round, and by the screw attached to it, expands the shanks and blades.

B. The joint which the Operator holds, as he turns round the screw-handle.

C. The shanks.

D. The blades.

The new Speculum Ani aut Vaginæ possesses a superiority over the old one in many particulars, both as a piece of Mechanism, and as conferring practical advantages in its application to Surgical purposes.

First.---The size of its Blades when shut, is at least two thirds smaller than the circumference of the common Speculum, and on this account can be introduced with more facility and less pain.

Secondly.---By its power of opening, the Blades can be expanded to a great extent, whilst the old Speculum always remains of the same Calibre from not possessing that power.

Thirdly.---When the blades are opened, they leave a large portion of the circumference of the Rectum or Vagina exposed to observation, whilst the old Speculum from not opening, covers the whole, and only enables the Surgeon to view the parts beyond the extremity of the instrument, which the new Speculum equally does.

Fourthly.---By turning the Blades of the new Speculum round, after they are introduced and opened, the Surgeon is enabled to examine the whole circle of the Rectum, an advantage the old Speculum does not confer, as it does not open and uncover any part of it.

Fifthly.---When the new Speculum is introduced and expanded, it supports itself in the situation it is fixed, and renders the assistance of a second Surgeon to hold it quite unnecessary, which the old Speculum requires, and as operations about the Rectum, &c. are more frequent in females than males, this instrument enables the Surgeon to spare their delicate feelings.

Sixthly.---If the new Speculum can be introduced beyond any Stricture of the Rectum, its power of opening will enable it to act as a very powerful Dilator of the Strictured part, and may supersede the employment of the Bougies in ordinary use.---These appear to be the advantages resulting from its superiority over the old Speculum as a piece of Mechanism.

Its applicability to some Surgical purposes, also demonstrates its superiority.---First, in case of tumours in the Rectum, Vagina or in the os uteri, the expansion of the new Speculum will enable the Surgeon to examine the Pedicle or Base more accurately, and to pass the Ligature around it more easily.---Secondly, In Fistula in ano, the Surgeon will be able to examine the course of the Fistula more correctly and perhaps to ascertain if the Fistula open into the Rectum, when this instrument has fully dilated it: he will also be able to introduce his finger more easily, and with more protection from the Bistoury; whilst, if he should divide an Artery that requires to be secured, he may probably be enabled to see it, and to secure it by a Ligature, which he could not do with the old Speculum; and in case he finds it impracticable to apply a Ligature, he would be enabled to apply the next best remedies, pressure and a Styptic, by placing the Styptic on the extremity of the bleeding vessel, and expanding the Blade of the Speculum placed upon it, by means of the screw.---Thirdly, in cases of internal Hæmorrhoids, in which it is necessary to excise a proportion of the relaxed membrane of the Rectum, this instrument enables its state to be examined in situ, previously to operation, and applications to be made to it after, if necessary.---Fourthly, In cases of that troublesome and painful ulceration about the Sphincter Ani, called "a crack," and which is formed between two of the Plicæ Ani, this instrument enables applications to be made, by which it will be probably healed without having recourse to the usual operation of excision. Fifthly, In cases of tumours situated as above mentioned, this instrument will enable the Surgeon to ascertain its nature, and apply appropriate remedies, and in cases of internal abscess in those parts to open the tumour more conveniently.

